# Tasting *Pseudomonas aeruginosa* Biofilms: Human Neutrophils Express the Bitter Receptor T2R38 as Sensor for the Quorum Sensing Molecule *N*-(3-Oxododecanoyl)-l-Homoserine Lactone

**DOI:** 10.3389/fimmu.2015.00369

**Published:** 2015-07-24

**Authors:** Susanne Maurer, Guido H. Wabnitz, Nadine A. Kahle, Sabine Stegmaier, Birgit Prior, Thomas Giese, Matthias Martin Gaida, Yvonne Samstag, Gertrud Maria Hänsch

**Affiliations:** ^1^Institute for Immunology, University of Heidelberg, Heidelberg, Germany; ^2^Institute of Pathology, University of Heidelberg, Heidelberg, Germany

**Keywords:** neutrophils, *N*-(3-oxododecanoyl)-l-homoserine lactone, quorum sensing, bitter receptor T2R38, lipid droplets, *Pseudomonas aeruginosa*

## Abstract

Bacteria communicate with one another via specialized signaling molecules, known as quorum sensing molecules or autoinducers. The *Pseudomonas aeruginosa*-derived quorum sensing molecule *N*-(3-oxododecanoyl)-l-homoserine lactone (AHL-12), however, also activates mammalian cells. As shown previously, AHL-12-induced chemotaxis, up-regulated CD11b expression, and enhanced phagocytosis of polymorphonuclear neutrophils. Circumstantial evidence concurred with a receptor for AHL-12, which has been elusive so far. We now investigated the bitter receptor T2R38 as a potential candidate. Although identified as a taste receptor, extragustatory cells express T2R38, for example, epithelial cells in the lung. We now detected T2R38 in peripheral blood neutrophils, monocytes, and lymphocytes. T2R38 is not only found on the cell membrane but also intracellular. In neutrophils, T2R38 was located in vesicles with characteristics of lipid droplets, and super-resolution microscopy showed a co-localization with the lipid droplet membrane. Neutrophils take up AHL-12, and it co-localized with T2R38 as seen by laser scan microscopy. Binding of AHL-12 to T2R28 was confirmed by pull-down assays using biotin-coupled AHL-12 as bait. A commercially available antibody to T2R38 inhibited binding of AHL-12 to neutrophils, and this antibody by itself stimulated neutrophils, similarly to AHL-12. In conclusion, our data provide evidence for expression of functional T2R38 on neutrophils, and are compatible with the notion that T2R38 is the receptor for AHL-12.

## Introduction

The quorum sensing molecule, *N*-(3-oxododecanoyl)-l-homoserine lactone (AHL-12), is produced by *Pseudomonas aeruginosa* and other Gram-negative bacteria. AHL-12 participates in the generation of virulence factors, and is particularly well studied in the context of biofilm formation (1–4). Of note, for AHL-12, a so-called “interkingdom-signaling” was reported, defined as activation of mammalian cells by bacterial signaling molecules (5, 6). Inhibition of various lymphocyte functions by AHL-12 was reported (7, 8), and was interpreted as a down-regulation of the immune response (9). On the other hand, we and others described activation of phagocytic cells, including enhancement of phagocytosis, increased expression of adhesion receptors, and induction of chemotaxis (10–12), leading to the presumption that recognition of AHL-12 might reinforce the local immune defense by preventing biofilm formation [reviewed in Ref. (13)].

AHL-12 is highly hydrophobic. For T cells and model lipid membranes, free diffusion through membranes was reported (14, 15). A nuclear receptor, PPARγ, was described in epithelial cells, although physical binding of AHL-12 to PPARγ has not yet been proven (16). In macrophages and neutrophils, the AHL-12 signaling pathway was compatible with that of a G-protein coupled receptor, whereas participation of toll-like receptors could be excluded (11, 13, 17–20), a receptor, however, has not yet been identified.

More recently, effects of AHL-12 on epithelial cells were described, such as enhanced ciliary movement and NO-production (21), indicating that AHL-12 induces activation of defense-related functions in these cells. On airway epithelial cells, the so-called bitter receptor T2R38 has been indicated as receptor for AHL-12, and there is evidence for a correlation between T2R38 receptor allotypes and susceptibility to airway infections (21–23).

The bitter receptor T2R38 belongs to a family of taste receptors. As the name implies, the receptor is sensing bitter tasting substances, a phenomenon that is thought to prevent the intake of bitter and potentially toxic substances [reviewed in Ref. (24–27)]. Bitter receptors were first described on cells of the taste buds in the oral cavity. Meanwhile, a wider distribution was reported, e.g., expression on epithelial cells of the airways, in the colon, in the brain, and in tumor cells as well (21, 28–30). The role of T2R38 in tissue outside the gustatory systems is still elusive, and so far, AHL-12 appears to be the first natural ligand for T2R38, which leads to the notion that T2R38 participates in “sensing of bacteria.” In that context, we now addressed the question whether T2R38 could also be the receptor for AHL-12 on neutrophils.

## Materials and Methods

### Human blood and human neutrophils

Blood from volunteers (mainly laboratory personal and students) was drawn into heparin-coated tubes (Sarstedt, Nümbrecht, Germany). Neutrophils were isolated by PolymorphPrep™(Axis-Shield, Oslo, Norway) and residual erythrocytes were removed by hypotonic lysis. The remaining cells were suspended in Hanks balanced salt solution (HBSS), containing 0.5% bovine serum albumin (BSA), at a final concentration of 5 × 10^6^ cells/ml. This procedure yielded 85–95% neutrophils as judged by cytofluorometry using CD66b as marker for neutrophils. Written informed consent was obtained from the volunteers and the ethic committee of the University of Heidelberg approved the procedure.

### Cell lines

U937 and HL-60 were purchased from ATCC. The cell lines were propagated in RPMI, containing fetal calf serum (10%), l-glutamine (1%), and penicillin-streptomycin (1%) (all purchased from Gibco, Eggenstein, Germany). The cells were used in their exponential growth phase. Differentiation of U937 was induced by phorbol ester (100 ng/ml); HL-60 was differentiated with DMSO (1.5%).

### Antibodies

The following antibodies to T2R38 were used: bs-86508-A488 [rabbit (rb)IgG, labeled with Alexa Fluor 488, Bioss, Woburn, MA, USA]; ab65509 (rb serum); ab 130503 (rb IgG) (both obtained from abcam, Cambridge, UK); sc-76108 (rb IgG), and sc-34294 (goat IgG) (Santa Cruz, Dallas, TX, USA). Isotype controls were rb IgG labeled with Alexa Fluor (bs-0295P-A488, Bioss, Woburn, MA, USA); rabbit IgG (b176094, abcam), or goat IgG (sc-3887, Santa Cruz). Rabbit serum was obtained from our animal facility. Secondary antibodies were FITC-labeled anti-rb IgG from goat (111-096-003, Dianova, Hamburg, Germany) or Alexa Flour 488-labeled anti-goat IgG from life technologies (A11055, Darmstadt, Germany). CD11b expression was determined using a FITC-labeled-antibody (IM0530, Beckman Coulter, Germany Krefeld). The antigen peptide, sc-34294P, used to raise the antibody to T2R38 (sc-34294), was purchased from Santa Cruz.

### Cytofluorometry

To determine the expression of surface receptors on neutrophils, whole blood (100 μl) or isolated neutrophils (10^7^/ml) were pre-incubated with an Fc-receptor blocking agent (Biolegend 422302, San Diego, CA, USA), then incubated with the respective antibodies or isotypic antibodies. In the whole blood assays, erythrocytes were lyzed using BD Facs Lysing solution (BD Biosciences, NJ, USA). Cells were subjected to cytofluorometry using FACSCalibur and CellQuest Pro software (Becton Dickinson, Heidelberg, Germany). For intracellular staining, the cells were treated with FACS Permeabilizing Solution 2 (BD Biosciences), pre-incubated with the Fc-receptor blocking agent and then incubated with anti-T2R38 (2 μg), as described above.

### Uptake of AHL-12 and inhibition experiments

Isolated neutrophils (1 × 10^6^/ml) in HBSS, containing 1% BSA and 0.1% sodium azide (“FACS buffer”) were incubated with AHL-FITC (Cayman Chemical, Ann Arbor, MI, USA) (50–100 μM) for 30 min at 4°C, and then subjected to cytofluorometry. For inhibition experiments, isolated neutrophils were washed with FACS buffer and incubated with antibodies to T2R38 for 30 min at 4°C, or with the antigen peptide (2–4 μg). AHL-FITC (100 μM) was added and after for 30 min at 4°C, fluorescence associated with the cells was measured.

### Detection of T2R38

A test kit (SEF811Hu) was purchased from Cloud Clone Corporation, Houston, TX, USA, and the test was carried out according to the protocol supplied.

### Gene expression analysis

DNA was isolated from 10^6^ purified neutrophils using the MagnaPure mRNA Isolation Kit I. mRNA was isolated with the MagnaPure-LC device. An aliquot of mRNA was reversely transcribed using AMV-RT and oligo-dT as primer (First Strand cDNA synthesis kit, Roche) according to the manufactures protocol. For PCR analysis, primer sets optimized for the LightCycler^®^ (RAS, Mannheim, Germany) were developed by and purchased from SEARCH-LC GmbH (www.Search-LC.com). The PCR was performed with the LightCycler^®^ FastStart DNA Sybr GreenI kit (RAS). To correct for differences in the content of mRNA, the calculated transcript numbers were normalized according to the expression of the housekeeping gene peptidylprolyl isomerase B (PPIB). Values represent number of transcripts per 1000 transcripts of PPIB.

### Western blot and pull-down assays

Isolated neutrophils or HL-60 (10^7^ cells) were lysed with RIPA buffer (Tris-buffered saline containing 1% Non-idet P-40, 0.5% sodium deoxycholate, 0.1% sodium dodecyl sulfate, 0.0004% sodium azide, 0.2M orthovanadate, and 0.5M phenylmethyl-sulfonyl fluoride). Of each sample, 25 μl were mixed with 5 μl 5× Laemmli-buffer and applied to a SDS-Gel (9%). Following blotting, the membrane was incubated with 5% milk powder in TBS, containing 0.1% Tween20. Of the antibody to T2R38 (sc-34294) a 1:1000 dilution was used, of antigen peptide sc-34294P 0.5/10 μl antibody. Secondary antibody was a POX conjugated mouse anti-goat IgG (#205-035-108, Jackson Immuno Research, Suffolk, UK, 1:20,000). Blots were developed using Amersham Prime Western Blotting detection Reagent (GE Healthcare, Freiburg, Germany).

For the pull-down assay, neutrophils or HL-60 (2 × 10^7^) were lysed in RIPA buffer (2 ml) and incubated overnight at 4°C with or without AHL-12-biotin (300 μM; Cayman Chemical). Then streptavidin sepharose beads (Cell signaling, Danvers, MA, USA) were added for 2 h at 4°C, then washed with PBS, and incubated with SDS loading buffer containing β-mercaptoethanol. The samples were applied to a 12% SDS-Gel. Silver staining was performed and Western blotting using sc-34294 and the respective antigenic peptide. Alternatively, to whole cell lysate, membrane proteins were extracted using the kit supplied by Calbiochem for transmembrane proteins (#71772-3, Merck, Darmstadt). Extracts of 4–5 × 10^7^ cells were used (neutrophils or HL-60, as indicated in the respective experiments).

### Isolation of lipid droplets

Essentially, the method described by Wan et al. (31) was used. In brief, 3 × 10^8^ U937 cells were suspended in 3 ml ice-cold disruption buffer [25 mM Tris-HCl pH 7.4, 5 mM EDTA, 1 mM EGTA, 0.2 mM PMSF, 50 μg/ml *N*-α-*p*-tosyl-l-lysin-chloromethyl-ketone (TLCK), 1 μg/ml leupeptin, 1 μg/ml pepstatin, 1 μg/ml aprotinin] and lysed by nitrogen cavitation (10 min, 800 psi, 4°C). The lysate was mixed with an equal volume of 1.08M sucrose in disruption buffer without TLCK, centrifuged for 30 min, 1000 g at 4°C, to eliminate nuclei and intact cells and then subjected to sucrose density centrifugation. After centrifugation (3.5 h, 34,000 rpm, 4°C), the lipid droplets were in the fraction with 0.54M sucrose. This fraction was diluted to 0.35M sucrose with 0.15M NaCl, overlaid with sucrose (0.27, 0.135 and 0M) for a second ultracentrifugation step (34,000 rpm, 4°C, 3.5 h). Lipid droplets were now in the fraction with 0.35M sucrose. Droplets could be recognized by fluorescence microscopy after having incorporated Nile red (see below). For Western blotting, the proteins were precipitated with methanol/chloroform, resuspended in Laemmli Buffer (95°C for 10 min) and applied to a 12% SDS-Gel. Silver staining and Western blotting was performed was described above.

### Laser scan microscopy

To assess binding of AHL-12 to cells and co-localization of T2R38, neutrophils were incubated with AHL-12-FITC (50 μM, 20 min at 4°C), placed on cover slips, and fixed with 2% PFA for 15 min. Then, anti-T2R38 [ab65509 (diluted 1:300) or sc-34294 (4 μg)] with the respective secondary antibodies (Alexa Fluor 488-labeled anti-rabbit IgG from donkey (bs-0295D-A488, Bioss); Alexa Fluor 488 Donkey anti-goat IgG (life technologies, Carlsbad, CA, USA) were added, and for comparison rabbit serum or goat IgG. The samples were mounted with Moviol (Sigma-Aldrich, St. Louis, MO, USA) and viewed by Laser Scan microscopy (LSM) (Nikon) using a 40× objective. To detect lipid droplets and the association of T2R38 with the droplets, neutrophils cells were placed on slides by cytospin, fixed with ice-cold methanol for 5 min and washed with PBS. Nile red (Sigma-Aldrich) was added (10 μg/ml 20 min, room temperature), and after washing Hoechst 33342 nuclear stain was applied (H3570, life technologies, Darmstadt, Germany) 1:10,000 for 10 min. The samples were embedded with Moviol. To visualize T2R38, 5% goat serum was used as blocking agent and anti-T2R38 (ab65509) in a dilution of 1:300 (for comparison, normal rabbit serum was used). After 30 min, the secondary antibody was applied for 30 min, then Nile red was added (10 μg/ml, 20 min). In addition, experiments with anti-T2R38 (sc-34294) were performed. In parallel, the antibody was added to the cells together with the peptide sc-34294P. Droplets were visualized by LSM and structured illumination microscopy (SIM) (D-Structured Illumination Microscopy), respectively.

### Phagocytosis

A commercially available kit (Glycotope Biotechnology, Heidelberg, Germany) with FITC-labeled bacteria was used according to the instructions supplied.

### Migration assays

Chemotaxis was determined using the BD Falcon HTS Fluoroblok 96-Multiwell Insert System with an insert PET membrane of 3 μm pore size. Isolated polymorphonuclear neutrophils (PMNs) were labeled with calcein. Cells (10^6^ cells/ml) were incubated for 15 min at 37°C with calcein (life technologies; 1 μg/ml), then washed with HBSS and seeded in a concentration of 2 × 10^5^/50 μl into the insert plate. AHL-12, PTU (100 μM), and for comparison HBSS or fMLP (10^−7^M) were added to the lower wells. After 2 h at 37°C, the insert plate was removed and cell-associated fluorescence was determined by a Perkin Elmer Wallac Victor 2 Microplate Reader using Wallac Workstation software.

For the “under-agarose” migration assay, cover slips (1.5 × 4 cm) were pretreated with 3M HCl and ethanol (95%). Following washing with water, gelatine (0.5% in RPMI) was applied as a thin film. The cover slips were air dried, then agarose (1% in RPMI) was added (3 ml per cover slip). When the agarose was solid, two holes were punched in a distance of 5 mm. Cells were placed in one hole (5 × 10^4^ in 50 μl), in the other the chemokine to be tested. After 2 h at 37°C, the cells were fixed with methanol for 30 min, the agarose was removed and the remaining cells were stained by Giemsa an examined microscopically.

## Results

### Expression of the bitter receptor T2R38 on neutrophils and monocytes

**Figure 1:**
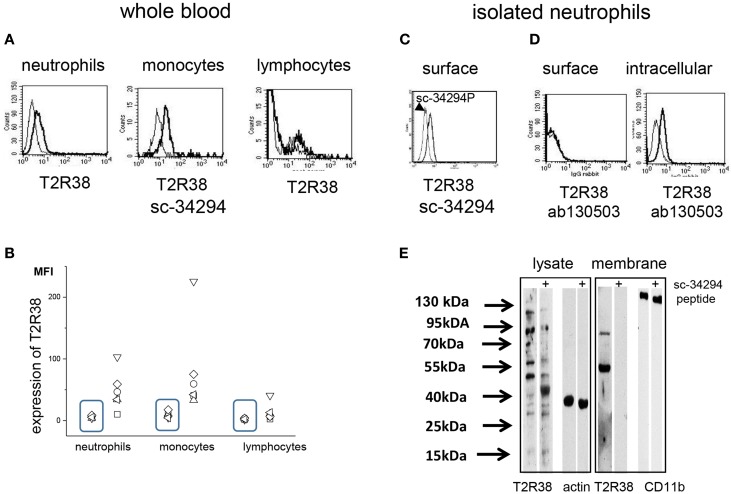
**Expression of T2R38 on peripheral blood cells**. **(A)** Cytofluorometry using the antibody sc-34294 (thick line) showed expression of T2R38 [the gates were set according to the cell populations identified by expression of CD66b (neutrophils), CD14 (monocytes), and CD3 (lymphocytes)]. For comparison goat IgG (thin line) was used. **(B)** T2R38 was determined by cytofluorometry on peripheral blood cells of seven individuals (each represented by a symbol) and mean fluorescence intensity (MFI) is shown. The rectangles contain the data for the antibody isotype binding. **(C)** T2R38 was also seen on isolated neutrophils (thick line) and binding of the antibody could be inhibited by the specific antigen peptide (thin line) **(D)** The antibody to T2R38 that recognizes the C-terminal domain of the receptor (ab130503) reacts only following permeabilization of the cells. **(E)** Western blot of neutrophil lysates with anti-T2R38 (sc-34294) revealed multiple bands. Intensity of bands with an apparent molecular weight of 90–100 kDa was reduced by the antigen peptide sc-34294p, as was the intensity of bands at 50–55 kDa (actin was used as loading control). Isolated membranes showed only two protein bands at approximately 90 and 50 kDa (CD11b was used as loading control). (Lysates and membranes were run on different gels, and are aligned here according to the molecular weight markers) (one of four blot is shown).

T2R38 was detected on neutrophils, monocytes, and to a lesser extent on lymphocytes in whole blood samples by cytofluorometry. Comparing samples of seven individuals, a considerably variation was seen (Figures 1A,B). Data obtained with the T2R38 antibody sc-34294 are shown, however, antibodies directed against other epitopes within the N-terminus of T2R38 (sc-67108; ab65509) gave essentially similar results (data not shown). For the antibody sc-34294, the antigenic peptide used to raise the antibody was available. This peptide inhibited binding of the antibody to the cells by 50% (Figure 1C). An antibody directed against an amino acid sequence of the C-terminus (ab130503) reacted only with permeabilized cells (Figure 1D). By Western blotting, T2R38 was detected in whole cell lysates and in cell membrane preparations. Specificity of the antibody binding was controlled by the antigenic peptide. In cell membranes, two bands with an apparent molecular weight of 90 and 50 kDa were detected, in the cell lysates multiple bands at 100, 90, 55, and 50 kDa, respectively (Figure 1E). The expected molecular weight of T2R38 is 38 kDa, but post-translational modifications and polymerization are described (for these experiments, sc-34294 was used).

T2R38 was also expressed by U937 and HL-60 cells (data not shown). Following differentiation of HL-60 by DMSO surface expression of T2R38 increased moderately (39.9% increase of the mean fluorescence intensity; average of three experiments).

**Figure 2:**
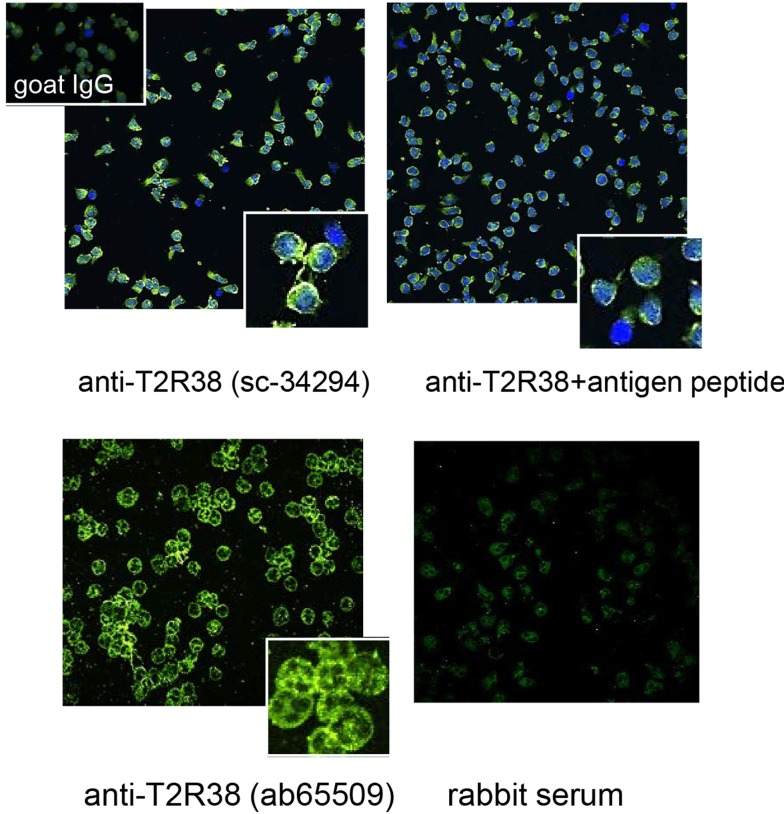
**Distribution of T2R38 on neutrophils: by laser scan microscopy, T2R38 was detected in neutrophils**. The upper panel shows an image with anti-T2R38 sc-34294 (green) and nuclei are stained in blue. Binding of the antibody was partly inhibited by the antigen peptide. The antibody ab65509 (lower panel) also detected T2R38 (green), in association with the membrane, and with a speckled pattern intracellular. The lower right panel shows the image obtained with rabbit serum instead of the antibody (images were taken with 40× objective; digital zooms of representative areas are shown in the insets).

**Figure 3:**
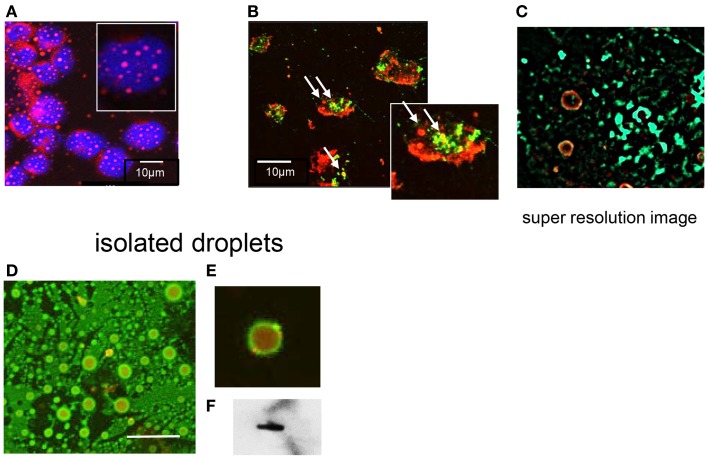
**Association of T2R38 with lipid droplets: (A) shows neutrophils stained with Nile red to visualize the lipid droplets; nuclei are in blue**. **(B)** With anti-T2R38 (green) and staining with Nile red, droplets containing T2R38 can be recognized within the cell (marked by arrows). **(C)** A SIM image of the area in the rectangle shows two lipids droplets with a distinct yellow staining of their membranes, indicative of a close association of T2R38 with the droplet membrane. **(D)** The images show isolated lipid droplets stained with Nile red and anti-T2R38; localization of T2R38 on the droplets membrane is seen especially in the zoomed image **(E)**. **(F)** shows a blot of isolated droplets with ab65509.

The distribution of T2R38 in neutrophils was analyzed by confocal LSM. Accumulation at the membrane was seen and a speckled pattern in the cytoplasm (images with two different antibodies are shown in Figure 2). The latter finding was compatible with the notion that intracellular T2R38 was associated with membranous vesicles and/or granules. Stimuli known to induce degranulation, including IL8, f-MLP, C5a, or phorbol ester, did not up-regulate surface expression of T2R38 when applied under conditions causing up-regulation of CD11b. Moreover, T2R38 was not released into the cell supernatant as determined by ELISA (data not shown). The failure to mobilize T2R38 makes its location in granules unlikely. Hence, we assessed association of T2R38 with lipid droplets. Neutrophils contain numerous lipid droplets visualized by incorporation of Nile red (Figure 3A). Confocal LSM indicated an association of T2R38 with droplets, particularly with their membrane (Figure 3B). To support this finding, we performed SIM. The circular morphology of the droplets could be resolved by this super-resolution microscopy, and T2R38 clearly was seen on the droplet membrane (Figure 3C). To verify the association of T2R38 with lipid droplets further, droplets were isolated by density centrifugation following the protocol by Wan et al. (31). Droplets of different sizes were seen, and again an association of T2R38 with the droplet membrane (Figures 3D,E). Lysates of the droplet fraction were positive for T2R38 as tested by blotting (Figure 3F).

### Effect of antibodies to T2R38 on AHL-12-mediated function

**Figure 4:**
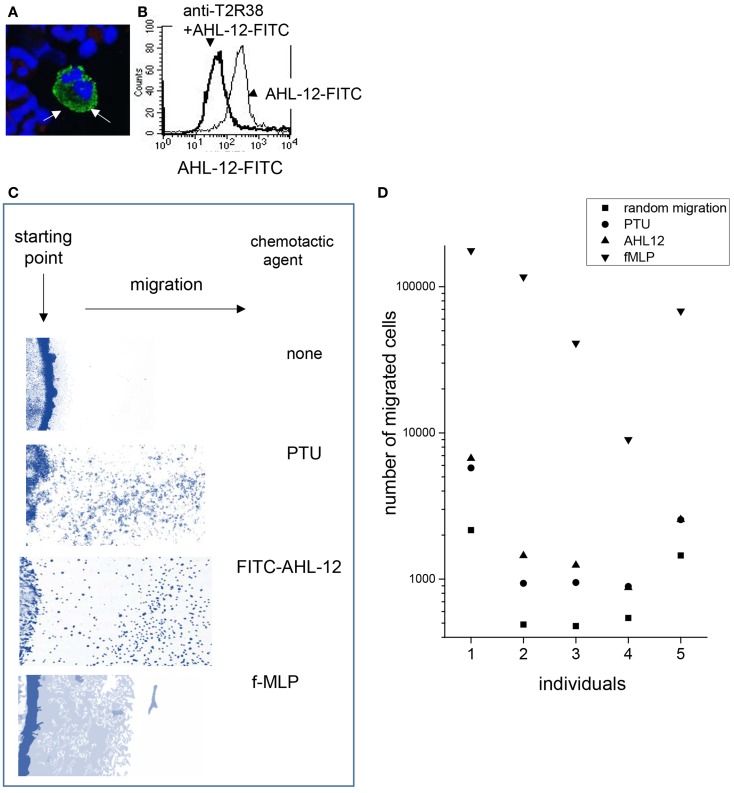
**Uptake of AHL-12-FITC by neutrophils and activation of cells: laser scan microscopy (A) and cytofluorometry (B) revealed binding of AHL-12-FITC to neutrophils (thin line)**. Binding could be inhibited by anti-T2R38 (sc-34294) (thick line) **(C)** AHL-12-FITC induced migration of neutrophils shown by “under-agarose” migration (cells are stained in blue), as did PTU. In comparison to f-MLP, fewer cells had migrated but had covered a greater distance. **(D)** Chemotaxis (here migration through porous membrane is shown; data with neutrophils of seven individuals are summarized) was induced by AHL-12, PTU, f-MLP, and compared to the random migration. Because endpoint was measured, the number of cell migrating toward f-MLP was considerably higher.

To answer the most pertinent question whether T2R38 might function as a receptor for AHL-12, the effect of antibodies to T2R38 on AHL-12 binding and AHL-12-induced neutrophil activation was tested. For these experiments, FITC-labeled AHL-12 was used. AHL-12-FITC binds to neutrophils, as seen by cytofluorometry and confocal LSM (Figures 4A,B). Binding of AHL-12-FITC could be inhibited by anti-T2R38 (sc-34294), on average by 35%, when 4 μg antibody were used per 1 × 10^6^ neutrophils/ml (mean of three experiments with cells of different individuals). With higher antibody concentrations, also the isotype control was inhibitory, precluding the correct interpretation of the data. The other antibodies to T2R38 (sc-67108 and ab65509) did not inhibit AHL-12 binding.

**Figure 5:**
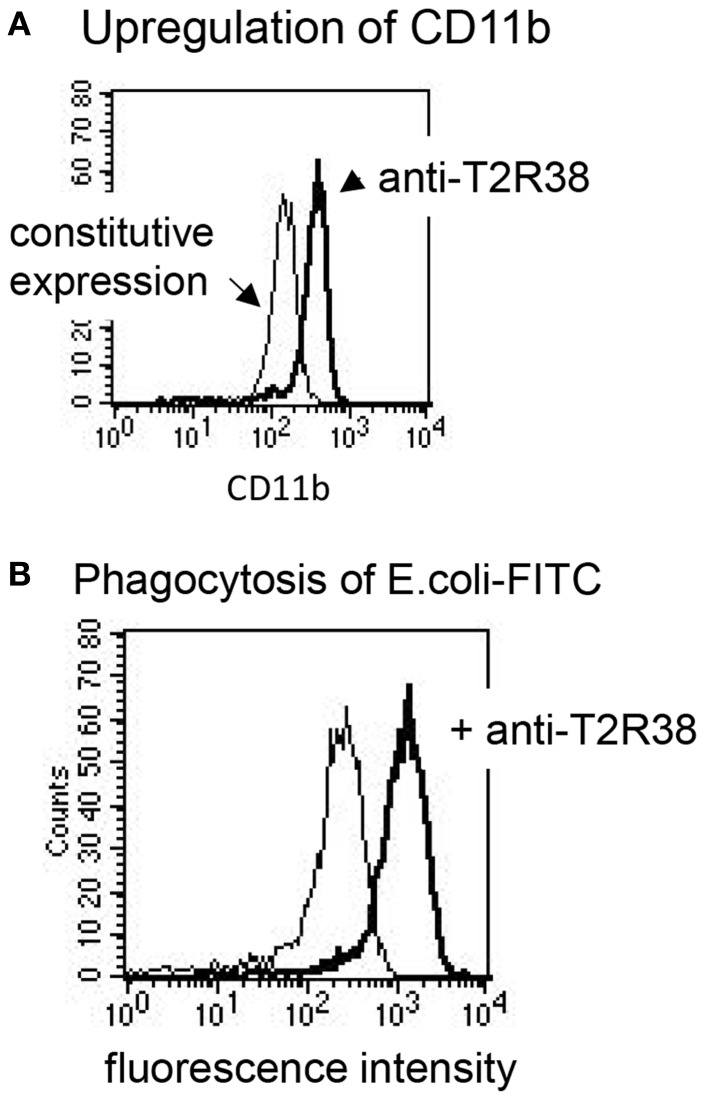
**Antibodies to T2R38 activate neutrophils**. **(A)** The antibody sc-34294-induced up-regulation of CD11b on neutrophils (thick line; the thin shows constitutive CD11b expression) and **(B)** it enhanced the phagocytosis of FITC-labeled bacteria, measured as increase of cell-associated fluorescence intensity (thick line; the thin line shows phagocytosis in presence of the isotype IgG).

Next, the effect of the antibody sc-34294 on AHL-12-mediated activation of neutrophils was assessed. Induction of chemotaxis was measured, enhancement of phagocytosis, and up-regulation of CD11b. Chemotaxis in response to AHL-12 was not inhibited, nor was migration toward PTU, which is widely used as ligand for T2R38, and which also induced chemotaxis of neutrophils (data based on two different methods, under-agarose migration and migration through a filter membrane, are shown in Figures 4C,D). Furthermore, the antibody did not inhibit the AHL-12 mediated up-regulation of CD11b nor the enhancement of phagocytosis. Rather, the antibody sc-34294 by itself activated neutrophils. Following antibody binding surface expression of CD11b was enhanced (on average by 1.7-fold; mean of three experiments), as was the oxidative burst elicited by phagocytosis of opsonized *E. coli* (on average by 3.5-fold) (examples in Figures 5A,B).

### Association of AHL-12 with T2R38 on neutrophils

**Figure 6:**
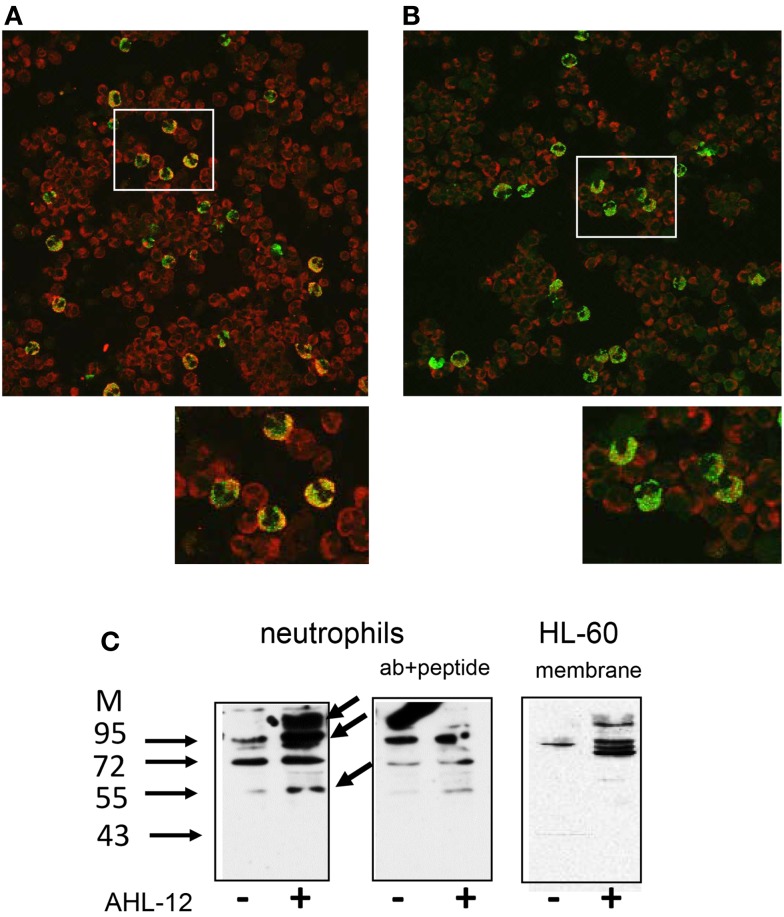
**Co-localization of AHL-12 and T2R38: neutrophils were incubated with AHL-12-FITC, placed on slides, fixed, and incubated with either (A) anti-T2R38 or (B) isotypic antibody**. Laser scan microscopy shows co-expression of T2R38 and AHL-12-FITC as “yellow cells.” The isotypic antibody shows some background staining, but the majority of cells remained “green.” Selected areas (white rectangles) were zoomed to show details. **(C)** A pull-down assays showed binding of AHL-12 to T2R38: AHL-12-biotin was incubated with neutrophils; then lysates were prepared, and incubated with streptavidin beads, and the eluates were subjected to SDS-PAGE and Western blotting using anti-T2R38 sc-34294 (lanes marked with +). In comparison to neutrophils having not been exposed to AHL-12-biotin (lanes marked with −), major bands were seen, at 100–130 kDa, and 55 kDa, the intensity of which could be reduced with the specific antigen peptide. Experiments with HL-60 showed essentially similar results. Here, membrane preparations are shown.

When neutrophils were incubated with limited amounts of AHL-12-FITC (50 μM) at 4°C and then incubated with anti-T2R38, confocal LSM revealed a close proximity of AHL-12 and T2R38 (Figures 6A,B), suggesting a physical association of AHL-12 and T2R38. “Pull-down” assays using biotin-labeled AHL-12 as bait were performed with lysates of neutrophils or HL-60. Cell lysates were incubated with AHL-12-biotin, and then with streptavidin beads. Eluates from the beads yielded numerous proteins. By Western blotting T2R38 could be identified with an apparent molecular weight of 100 and 60 kDa (data for neutrophils and for HL-60 are shown Figure 6C). When the pull-down assay with AHL-12-biotin was carried with membrane protein fractions, T2R38 could be detected again by Western blotting (Figure 6C).

## Discussion

Our data provide evidence for an extragustatory expression of the bitter taste receptor T2R38 on neutrophils, monocytes, and myeloid cell lines. By a variety of methods and with antibodies directed against different epitopes, intracellular expression as well as expression on the cell membrane was seen. At difference with the predicted molecular weight of 38 kDa, proteins with higher apparent molecular weight were detected, presumably the result of post-translational modifications and polymerization of the receptor, as it had been described for T2R38 in epithelial cells (32).

Surface expression of T2R38 varied among individuals, as did the number of gene transcripts. We presume a genetic predisposition, because multiple testing revealed individuals expressing little T2R38 and others expressing moderate levels (own unpublished data). The histograms obtained by cytofluorometry revealed a more or less symmetric peak, indicating that T2R38 expression was not restricted to a subpopulation. The number of gene transcripts was rather low. Absent or marginal gene transcription – even for highly expressed proteins – is not unusual for neutrophils, because in these cells a great majority of proteins is produced during maturation only, and are then stored in intracellular compartments (33, 34).

Well-characterized storage compartments of neutrophils are granules, which are generated as the precursor cells mature, and which enclose proteins as they are synthesized [reviewed in Ref. (34, 35)]. Release of proteins from those compartments is accomplished by a number of mediators, including IL-8, f-MLP, C5a, or phorbol ester (36). Because these compounds failed to mobilize T2R38, we looked for other storage compartments and found T2R38 in association with vesicles having the properties of lipid droplets. Lipid droplets were originally described as lipid storage sites, but more recent studies described them as functional organelles that actively participate in lipid turnover, cellular signaling, and membrane trafficking (37, 38). As far as we know, storage in or association with vesicles – lipid droplets or others – has not been investigated for T2R38 in its usual habitat – that is, in cells of the taste buds – or in epithelial cells. Thus, the question remains whether or not storage of T2R38 in vesicles is typical for neutrophils or whether it is a general feature of T2R38 (our own not yet published data point to the latter).

Having established that T2R38 is present in neutrophils, the question was addressed whether it is the receptor for AHL-12 on these cells. LSM showed a co-localization of AHL-12 and T2R38, and the “pull-down-assay” with biotin-labeled AHL-12 indicated a physical interaction of AHL-12 with T2R38, which implies that T2R38 is indeed the receptor for AHL-12 on neutrophils.

To link T2R38 to AHL-12 signaling, the effect of antibodies to T2R38 on AHL-12-induced functions was tested. Only one of the antibodies (sc-34294) inhibited binding of AHL-12 to some extent, but not entirely. A likely explanation is the hydrophobic nature of AHL-12, which allows attachment to cells and probably also partition into the membrane in a receptor-independent manner. That the other antibodies – though binding to the cells – did not block potential AHL-12 binding sites is in line with data reported for ab65509 by others in another setting (32). As anticipated, these antibodies did not inhibit AHL-12-mediated activation of neutrophils.

The antibody sc-34294, which inhibited AHL-12 binding, stimulated neutrophils. The antibody by itself up-regulated CD11b cell surface expression, and it enhanced phagocytosis, two functions that are also induced by AHL-12. Thus, T2R38 is a functional receptor on neutrophils, and according to the co-localization data, AHL-12 is the most likely ligand.

Expression of T2R38 on neutrophils fits the concept that the cells “sense” bacterial infection, and up-regulate defense-relevant actions in consequence. In that, T2R38 qualifies as pathogen-associated molecular pattern recognition receptor. Because bacteria produce and release AHL-12 as an autoinducer, crucially required for biofilm formation, neutrophils will be attracted to the infectious site before or concurrent with biofilm formation. Because planktonic bacteria are in general more susceptible to host defense mechanisms compared to bacteria organized as biofilm, the infection can be cleared. Valid objections to this notion are that micromolar concentrations of AHL-12 are required to activate neutrophils – roughly 100- to 10,000-fold more than for established chemokines – and that the weak to moderate surface expression of T2R38 questions the effectiveness of an AHL-12–T2R38 interaction. However, according to our experience, AHL-12 is not very stable under cell culture conditions, meaning that the abundance of active molecules might be lower than the calculated input. In addition, local concentrations of AHL-12 could be rather high, as data using supernatants of biofilm-forming *P. aeruginosa* imply (20). Without question, the biological relevance of our finding is still under scrutiny.

Our data, however, establish for the first time extra gustatory expression of T2R38 on cells of the myeloid lineage, and are compatible with the notion that T2R38 functions as a receptor for AHL-12 on neutrophils as a novel means of recognizing of bacterial components by the host defense.

## Author Contributions

All authors approved the final version of the manuscript, and contributed substantially to the study: SM did the cytofluorometry experiments, GW and SS the laser scan microscopy and SIM, NK the initial experiments on neutrophil activation and the uptake studies. BP performed the functional assays (chemotaxis and phagocytosis), TG did the quantitative PCRs, SM, MG, YS, GW, and GH did the data analysis and wrote the manuscript.

## Conflict of Interest Statement

The authors declare that the research was conducted in the absence of any commercial or financial relationships that could be construed as a potential conflict of interest.
